# Increased expression of caspase 1 during active phase of connective tissue disease

**DOI:** 10.7717/peerj.7321

**Published:** 2019-07-22

**Authors:** Rentian Cai, Qiongqiong Wang, Gongmin Zhu, Liying Zhu, Zhen Tao

**Affiliations:** 1 Department of Infectious Diseases, Nanjing First Hospital, Nanjing Medical University, Nanjing, Jiangsu, China; 2 Nuclear Medicine Department, Nanjing First Hospital, Nanjing Medical University, Nanjing, Jiangsu, China

**Keywords:** Caspase 1, Caspase 4, Caspase 5, Connective tissue disease

## Abstract

Key factors of pyroptosis play an important role in the inflammatory response to connective tissue disease (CTD). However, information on active and stable stages of CTD is scarce. To distinguish the differences of concentrations of C-reactive protein (CRP), caspase 1, caspase 4, caspase 5 and sCD14 in plasma between the patients with active and stable stages of CTD. A cohort study was conducted to recruit patients diagnosed with CTD of active phase and stable phase as well as health control. These data included the analysis of the concentration of sCD14, caspase 1, caspase 4 and caspase 5 in peripheral plasma by ELISA. The Wilcoxon rank-sum test was used to compare the two groups. The sex ratio and ages of the three groups were not different statistically. The concentrations of sCD14, caspase4 and caspase5 of plasma in the CTD of active phase and the stable phase as well as the health control. The concentration of caspase 1 in active phase of CTD (470.19 [422.33–513.14] pmol/L) was significantly higher than that in stable group (203.95 [160.94–236.12] pmol/L) and healthy control (201.65 [191.11–240.35] pmol/L] pmol/L) (*p* < 0.001, both), but there was no significant difference between stable group and healthy control (*p* = 0.2312). Similarly, the concentration of CRP in the active phase of CTD (8.96 [3.06–20.28] mg/L) was significantly higher than that in the stable group (3.00 [1.30–11.40] mg/L) and the healthy control (3.70 [2.30–4.73] mg/L) (*p* = 0.0013, *p* = 0.0006, respectively), but there was no significant difference between the stable group and the healthy control (*p* = 0.3205). However, there were no significant differences in the concentration of sCD14, caspase 4 and caspase 5 in the active phase of CTD and the stable group as well as the health group. Consequently, the patients of the active phase of CTD showed increased expression of caspase 1.

## Introduction

Connective tissue disease (CTD) is a common clinical disease, including rheumatoid arthritis, systemic lupus erythematouses, dermatomyositis, Sjogren’s syndrome, undifferentiated CTD and so on. Different types of CTDs have similar clinical manifestations. The pathogenesis is similar that is the attack of autoantibodies on connective tissue (Akashi, Saegusa & Morinobu, 2015; Murakami & Mimori, 2019). In addition, disordered release of inflammatory factors and intestinal flora dysregulation contribute to the pathogenesis of CTD (Hasegawa & Takehara, 2012; Nakken, Bodolay & Szodoray, 2015; Talotta et al., 2017).

Previous studies have shown that caspase 1 plays an important role in the inflammatory response to CTD. For example, patients with Sjogren’s syndrome (Niu et al., 2015; Vakrakou et al., 2018), rheumatoid arthritis (Choulaki et al., 2015; Guo et al., 2018; Yang et al., 2016), and systemic lupus erythematouses (Zhang et al., 2016), dermatomyositis and polymyositis (Yin et al., 2016) have the feature of caspase 1-mediated release of inflammatory factors. Furthermore, CTD patients manifested as intestinal flora dysregulation such as increased endotoxin (Talotta et al., 2017). Endotoxin can bind to receptor CD14 (Hailman et al., 1994), which can activate caspase 4 and caspase 5 (Baker et al., 2015; Lagrange et al., 2018; Shi, Gao & Shao, 2016). These three enzymes can induce the pyroptosis of immune cells, promote the release of intracellular contents, cause fever and other clinical manifestations (Bergsbaken, Fink & Cookson, 2009; Shi, Gao & Shao, 2016).

However, there are active and stable stages of CTD. The clinical manifestations of active stage are typical, while there are no special clinical manifestations of stable stage. Previous studies have not focused on the activity and stability of CTD related inflammatory factors and indicators of intestinal flora dysregulation.

Therefore, this study intends to reflect on the key factors of the pathogenesis of CTD from patients’ plasma, including caspase 1, caspase 4 and caspase 5 which play a key role in the secretion of inflammatory factors, and sCD14 which reflects the intestinal flora dysregulation.

## Materials & Methods

### Study subjects

All the patients in this group were enrolled from May 2017 to May 2018 in Nanjing First Hospital. Inclusion criteria: patients diagnosed with CTD; Exclusion criteria: patients with HIV, HBV, HCV and tuberculosis infection and long-term corticosteroids use; Healthy controls with CTD, HIV-1, HBV, or HCV infection or with corticosteroids were excluded. The case groups were divided into active phase and stable phase of CTD. The stable phase of CTD was defined as presenting no fever, synovitis, serositis, myositis. Conversely, the active phase of CTD was considered if not defined by the above criteria. The patients’ peripheral plasma was frozen at −80 °C for experiment, and the clinical data were recorded.

### Ethics approval and consent to participate

The study protocols were approved by the Research Ethics Committee of Nanjing First Hospital. This committee waived the need for written informed consent from the participants because the study was retrospective, anonymous, and only used currently existing data.

### Measurements of factors in peripheral plasma

The reagents for the detection of caspase 1, caspase 4, caspase 5 and sCD 14 in peripheral plasma were purchased from Shanghai Lanpaibio Company. The detection of these factors was used by Bio-RAD EILISA equipment. The experimental operation was carried out according to the kit instructions. The samples were diluted five times, then stained and eluted. OD value data were read on the enzyme labeling instrument. Concentration was obtained according to the concentration standard curve, then multiplied by 5. That was the original concentration. C-reactive protein (CRP) was tested by turbidimetric inhibition immunoassay.

### Statistical analyses

For normal distribution and homogeneous variance data are shown as mean ± standard deviation. Student t test is used for comparison between two independent samples. For data that does not meet the above conditions, data distribution is shown as median (interquartile range) and the Mann–Whitney *U* test is used to compare statistical significance between groups. The chi-square (*χ*^2^) test is applied to analyze the categorical variables. All statistical analyses are performed with SPSS, version 16.0 (SPSS, Inc., Chicago, IL, USA), and GraphPad Prism, version 5.0, software (GraphPad Software, San Diego, CA, USA).

## Results

### Characteristics of the study population

Patient characteristics are shown in Table 1.

**Table 1:** General characteristics of the study subjects.

Variable	Active phase of CTD (*n* = 46)	Stable phase of CTD (*n* = 39)	Healthy control (*n* = 38)	*p* value
Age ($\overline{X}$± s, year)	66.09 ± 12.91	64.33 ± 14.84	63.66 ± 5.47	0.624
Sex (% male)	17(36.96)	15(38.46)	17(44.74)	0.752
Rheumatoid arthritis	13	6	–	0.407
ANCA-associated vasculitis	1	0	–	
SLE	1	0	–	
Sjogren’s syndrome	2	1	–	
Takayasu arteritis/Temporal arteritis	1	1	–	
Rheumatic polymyalgia	0	1	–	
Dermatomyositis	0	2	–	
Mixed CTD	1	0	–	
Undifferentiated CTD	27	28	–	

**Notes.**

$\overline{X}$± s mean ± standard deviation CTD connective tissue disease SLE systemic lupus erythematosus

Three groups of subjects: active phase of CTD, stable phase of CTD and healthy control, most of them are middle-aged and elderly, mainly female, the age of each group is 66.09 ± 12.91, 64.33 ± 14.84, 63.66 ± 5.47.There is no statistical difference in sex ratio and age difference between the two groups (Table 1).

### The concentration of caspase 1 in active phase of CTD is higher

**Figure 1:**
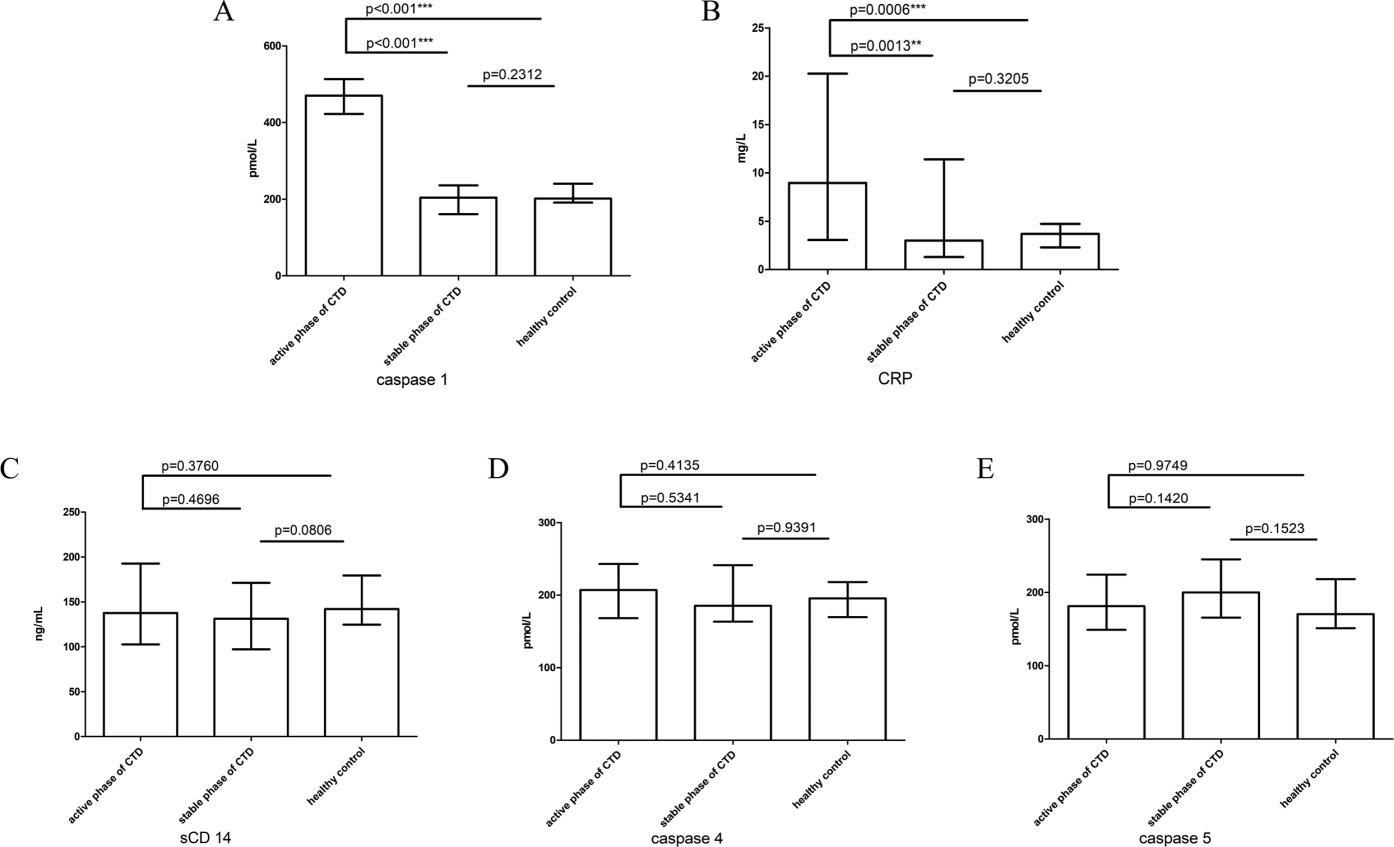
The concentration of detected indicators in peripheral plasma. The concentration of detected factors in peripheral plasma. The concentration of caspase 1 (A), CRP (B), sCD14 (C), caspase 4 (D), caspase 5 (E) in the groups of active phase of CTD, stable phase of CTD and healthy control. note: CRP, C-reactive protein; CTD, connective tissue disease; **, *P* < 0.01; ***, *P* < 0.001.

The concentrations of caspase 1 in peripheral plasma of the three groups are 470.19 [422.33–513.14] pmol/L, 203.95 [160.94–236.12] pmol/L and 201.65 [191.11–240.35] pmol/L, respectively. The concentration of caspase 1 in CTD active group is significantly higher than that in stable group, but there is no significant difference between the stable group and the healthy control (Fig. 1A).

In addition, as a non-specific index of inflammatory response, CRP was detected in plasma. The concentrations of CRP in peripheral plasma of the three groups are 8.96 [3.06–20.28] mg/L, 3.00 [1.30–11.40] mg/L and 3.70 [2.30–4.73] mg/L, respectively. It is found that the CRP concentration in the CTD active group is significantly higher than that in the stable group, while there is no significant difference between the stable group and the healthy control. The results are similarly to caspase 1 (Fig. 1B).

### No significant differences in concentrations of sCD14, caspase 4 and caspase 5 between active phase and stable phase of CTD

CD14 is an endotoxin receptor. The soluble CD14 (sCD14) in plasma can indirectly reflect endotoxin level in plasma. We measured plasma sCD14 concentration of 137.62 [102.59–192.62] ng/mL, 131.28 [97.26–171.25] ng/mL and 142.12 [124.58–179.41] ng/mL in the three groups, respectively. The results showed that there was no significant difference in the three groups (Fig. 1C).

Endotoxin can activate caspase 4 as well as caspase 5 and induce pyroptosis of cells. We measured plasma concentrations of the two markers in the three groups, 207.01 [168.31–243.10] pmol/L, 185.34 [163.48–241.25] pmol/L, and 195.54 [169.77–217.99] pmol/L, as well as 290.56 [221.19–410.68] pmol/L; 200.06 [165.74–245.74-245.09] pmol/L and 170.47 [151.31–151.31-218.06 ] pmol/L, respectively. We found that the concentrations of caspase 4 and caspase 5 had no difference in the three groups (Figs. 1D & 1E).

## Discussion

Our results showed that the concentrations of caspase 1 as well as CRP in the active group were significantly higher than that in the stable group of CTD.

Our study complemented previous studies that showed that caspase 1 level in CTD inclduing rheumatoid arthritis, SLE, Sjogren’s syndrome, dermatomyositis and so on, whether in tissues or in plasma, increased significantly (Cascao et al., 2012; Guo et al., 2018; Monteith et al., 2018; Vakrakou et al., 2018; Yin et al., 2016; Zhai et al., 2018). In patients with SLE, activated caspase 1 in macrophage cleaved Rab39a to promote the production of immune complexes (Monteith et al., 2018). In patients with rheumatoid arthritis, active caspase 1 cleaved IL-1β precursors and IL-18 precursors to IL-1β and IL-18 to induce inflammation, respectively (Cascao et al., 2012). However, another study found that caspase 1 expression was decreased in patients with rheumatoid arthritis, but the activity of caspase 1 was significantly increased, which only promotes the production of IL-18, not IL-1β (Yang et al., 2016). In Sjogren’s syndrome, the concentration of caspase 1 in gingival crevicular fluid increased significantly, while the concentration of caspase 1 in peripheral plasma decreased, but the concentration of IL-1β in both parts increased significantly (Ozcaka et al., 2018). The reason for this phenomenon may be that previous studies did not divide CTD into active and stable stages. The former is characterized by inflammatory activity, while the latter is characterized by relatively stable condition. CPR is a nonspecific inflammatory marker. Our study showed that inflammatory activity was observed in the active phase of CTD, but not in the stable phase.

In fact, damage associated molecular patterns (DAMPs), including CTD-related DAMPs, can activate caspase 1, promote the production of inflammatory cytokines including IL-1 and IL-18 in immune cells, such as lymphocytes and monocytes as well as macrophages, and induce cells death, and inflammatory cytokines are disordered released to induce inflammation (Schroder & Tschopp, 2010). Therefore, caspase 1 might be used as one of the therapeutic targets for active phase of CTD. A study suggested that caspase 1 inhibitor was effective against CTD (Vande Walle et al., 2014). Our study suggested that caspase 1 inhibitors might be more effective in patients with active phase of CTD than stable phase.

In addition, we used sCD14, caspase 4 and caspase 5 to evaluate intestinal flora dysregulation of patients with CTD. Our study suggested that there was no significant difference between the active group and the stable group and the healthy control group. It suggested that intestinal flora dysregulation might not play an important role in the pathogenesis of CTD, which was different from previous studies. Previous studies showed that there was intestinal flora dysregulation in patients with CTD (Talotta et al., 2017). Some studies had found that some CTD, including SLE, systemic sclerosis and Sjögren’s syndrome had intestinal microbiome unbalance (Andreasson et al., 2016; De Paiva et al., 2016; He et al., 2016; Hevia et al., 2014; Volkmann et al., 2016). But there were no available data concerning the role of the microbiome in undifferentiated CTD and mixed CTD (Talotta et al., 2017). Our findings showed that intestinal flora dysregulation might not play an important role in CTD. For the contradictory conclusion, further research is needed to clarify the causes of different results.

There are some defects in our study. First, the number of cases is relatively small, and secondly, CTD is not classified according to specific diseases. If there are enough research subjects, we can classify the patients according to the types of CTD, which will make our research more concrete. This will be further improved in future research.

## Conclusion

The patients of the active phase of CTD showed increased expression of caspase 1, but did not show a concentration of sCD14, caspase 4 and caspase 5.

##  Supplemental Information

10.7717/peerj.7321/supp-1 Data S1 Clinical data and ELISA applied for data analyses and preparation for Table 1 and Figure 1

10.7717/peerj.7321/supp-2 Data S2 Codebook of raw data

## Additional Information and Declarations

### Data Availability

The following information was supplied regarding data availability: Raw data is available as a Supplemental File.
