# Supplementary material for: Increased expression of caspase 1 during active phase of connective tissue disease
Source: PeerJ. 2019 Jul 22;7:e7321. doi: 10.7717/peerj.7321 (PMC6657674; doi:10.7717/peerj.7321)
Supplement: Data S2 [file peerj-07-7321-s002.docx]

codebook of raw data:

group:

1: active phase of CTD

2: stable phase of CTD

3: healthy control
